# Serum Uric Acid Is Associated with Incident Chronic Kidney Disease in Middle-Aged Populations: A Meta-Analysis of 15 Cohort Studies

**DOI:** 10.1371/journal.pone.0100801

**Published:** 2014-06-24

**Authors:** Ping Zhu, Yan Liu, Lu Han, Gang Xu, Jian-min Ran

**Affiliations:** 1 Department of Endocrinology and Metabolism, Guangzhou Red Cross Hospital, Medical College of Jinan University, Guangzhou, China; 2 Department of Nephrology, Guangzhou Red Cross Hospital, Medical College of Jinan University, Guangzhou, China; 3 Department of Medical Statistics and Epidemiology, School of Public Health, Sun Yat-sen University, Guangzhou, China; Mario Negri Institute for Pharmacological Research and Azienda Ospedaliera Ospedali Riuniti di Bergamo, Italy

## Abstract

**Background:**

Mounting evidence indicates that elevated serum uric acid may increase the incidence of chronic kidney disease (CKD). Our goal was to systematically evaluate longitudinal cohort studies for the association of serum uric acid levels and incident CKD.

**Methods:**

We searched electronic databases and the reference lists of relevant articles. The primary outcome was incident CKD, which was defined as an eGFR less than 60 mL/min/1.73 m^2^ at the follow-up examination. Study-specific risk estimates were combined using random-effects models. The included studies were stratified into subgroups, and meta-regression analyses were performed.

**Results:**

Fifteen unique cohorts with a total of 99,205 individuals and 3,492 incident CKD cases were included. The relative risk of CKD was 1.22 (95% CI 1.16–1.28, I^2^ = 65.9%) per 1 mg/dL serum uric level increment. This positive association was consistently observed in subgroups stratified according to most of the study-level characteristics. The observed positive association was more pronounced among group with a mean age <60 years (RR 1.26, 95% CI 1.21–1.31), and low-level heterogeneity was observed in the findings for this age group (I^2^ = 46.4%, P = 0.022). However, no association was observed among studies with a mean age≥60 years (RR 1.04, 95% CI 0.96–1.13), and no evidence of heterogeneity was evident among the studies (I^2^ = 0%, P = 0.409). This mean age-related difference in the association between serum uric acid levels and CKD was significant (P = 0.004). The sensitivity analysis results were consistent when the analyses were restricted to studies that controlled for proteinuria and metabolic syndrome.

**Conclusions:**

Our meta-analysis demonstrated a positive association between serum uric acid levels and risk of CKD in middle-aged patients independent of established metabolic risk factors. Future randomized, high-quality clinical trials are warranted to determine whether lowering uric acid levels is beneficial in CKD.

## Introduction

The incidence of chronic kidney disease (CKD) is high and increasing worldwide, particularly in developing countries [1]. For example, in China, 119.5 million adults 18 years or older (10.8% of the adult population) have CKD [2]. Therefore, the detection and prevention of CKD are important issues in health care.

Increasing evidence supports a significant correlation between serum uric acid levels and the development of CKD. A recently published paper [3] reviewed the relationship between hyperuricemia and CKD based on longitudinal observational and interventional studies and raised the possibility of uric acid as a potential therapeutic target to prevent the onset of CKD. As elevated serum uric acid is a proposed risk factor for cardiovascular disease and other established cardiovascular risk factors, such as diabetes [4], hypertension [5], and metabolic syndrome [6], whether serum uric acid is an independent risk factor for the development of CKD or is merely an incidental finding related to the severity of CKD remains unknow [7], [8], [9], [10], [11]. Although one previous meta-analysis reported a positive association between serum uric acid levels and an increased risk of CKD [7], the evidence was limited due to unexplained, moderate heterogeneity across the included studies. Additionally, possible confounding by other factors needs to be explored to firmly establish the potential role of uric acid in CKD. Therefore, given the recent accumulation of evidence and intervention studies [12], we conducted a meta-analysis of longitudinal cohort studies to quantify the nature and magnitude of the association between serum uric acid levels and incident CKD and to measure this association according to the study design and population characteristics.

## Subjects and Methods

### Search Strategy

This meta-analysis follows PRISMA guidelines (Checklist S1) [13]. Relevant studies were identified by searching PubMed, EMBASE, the Cochrane library, China CNKI, and Wanfang databases for all articles published until March 2014 with the following text and key words in combination (both as MeSH terms and text words): “uric acid”, “hyperuricemia”, “urate”, “chronic kidney disease”, “chronic renal disease”, “chronic renal insufficiency”, “chronic kidney failure”, “kidney disease”, “risk factors”, “prospective studies”, “retrospective studies”, “cohort studies”, and “follow-up studies”. Additionally, we reviewed the reference lists of the retrieved papers and recent reviews. No language restrictions were applied. When necessary, we corresponded with the investigators of the original studies.

### Study Selection

We first performed an initial screen of the titles and abstracts. A second screen was based on a full-text review. We included studies that measured the serum uric acid levels at baseline and documented incident CKD at follow-up. Incident CKD was defined as individuals who were free of CKD at baseline (eGFR ≥60 mL/min/1.73 m^2^) but experienced a decline in their eGFR to less than 60 mL/min/1.73 m^2^ at a follow-up examination. For inclusion, the publications were required to contain estimates of the relative risk (RRs) and 95% confidence intervals (CI). We excluded case-control and cross-sectional studies to reduce the bias from pre-existing CKD on serum uric acid levels (reverse causation). In cases of multiple publications reporting the same dataset, the most up-to-date or comprehensive information was used.

### Data Extraction and Quality Assessment

When available, the following data were extracted: author name, publication date, country of origin, characteristics of study population, baseline mean age and serum uric acid level, duration of follow-up, number of cases and total participants, adjustment for potential confounders in a multivariate analysis, and estimates of association (hazard ratios [HR] or odds ratio [OR] and 95% CI). For studies conducted using different subsamples of the same cohort, we separately extracted data from the multiple publications. The study quality was assessed by reporting the key components of each cohort [14], including the characteristics of the study population, assessments of the exposure and outcome, length of follow-up, and statistical control for potential confounding factors. Two investigators (Ping Zhu and Jian-min Ran) performed the literature search, data extraction, and quality assessment. Disagreements were reconciled through discussion.

### Statistical Analysis

The RR with a 95% CI was adopted as the common measure of association across the studies. Most studies (data extracted from conversations with three of the authors were also included) reported the RRs of CKD for each 1 mg/dL or standard deviation of higher serum uric acid levels. For two studies [10], [15] that analyzed the serum uric acid level as a categorical variable instead of as a continuous variable, we calculated the corresponding RRs for CKD for a 1 mg/dL increase in the serum uric acid level as previously described [16], [17], [18]. Briefly, when the participants were divided into four equally sized groups, we used the reported standard deviation to estimate the 12.5th and 87.5th percentiles of serum uric acid under the assumption of normality for serum uric acid. Then, the log RRs were divided by the difference of these two values to estimate the effect of a 1 mg/dL change in serum uric acid. The homogeneity of the RRs across the studies was assessed with the Q statistic at P<0.10. The I^2^ statistic, which is a quantitative measure of inconsistency across studies [19], was also calculated. The combined risk estimates were computed using a random-effects model [20].

Subgroup analysis was explored using meta-regression to examine the statistical significance of the difference in the RRs based on the study location (Asian or non-Asian), gender (men or women), mean serum uric acid level (≥5.5 or <5.5 mg/dL), mean age (≥60 or <60 years), mean follow-up duration (≥5 or <5 years), measure of association (OR or HR), and source of the subjects (health check or non-health check) given that these factors can contribute to study heterogeneity. We performed sensitivity analyses that were restricted to studies with additional adjustment for proteinuria, eGFR (or serum creatinine, SCr), or metabolic parameters. Additionally, we assessed the influence of individual studies on the pooled results by omitting each study individually and reevaluating the combined estimates of the remaining studies.

Publication bias was assessed using the Begg’s and Egger’s tests [21], [22]. We used STATA version 11.0 (Stata Corp) for all analyses. P<0.05 was considered statistically significant except for the test of publication bias, which had a level of significance of P<0.10.

## Results

### Literature Search

**Figure 1:**
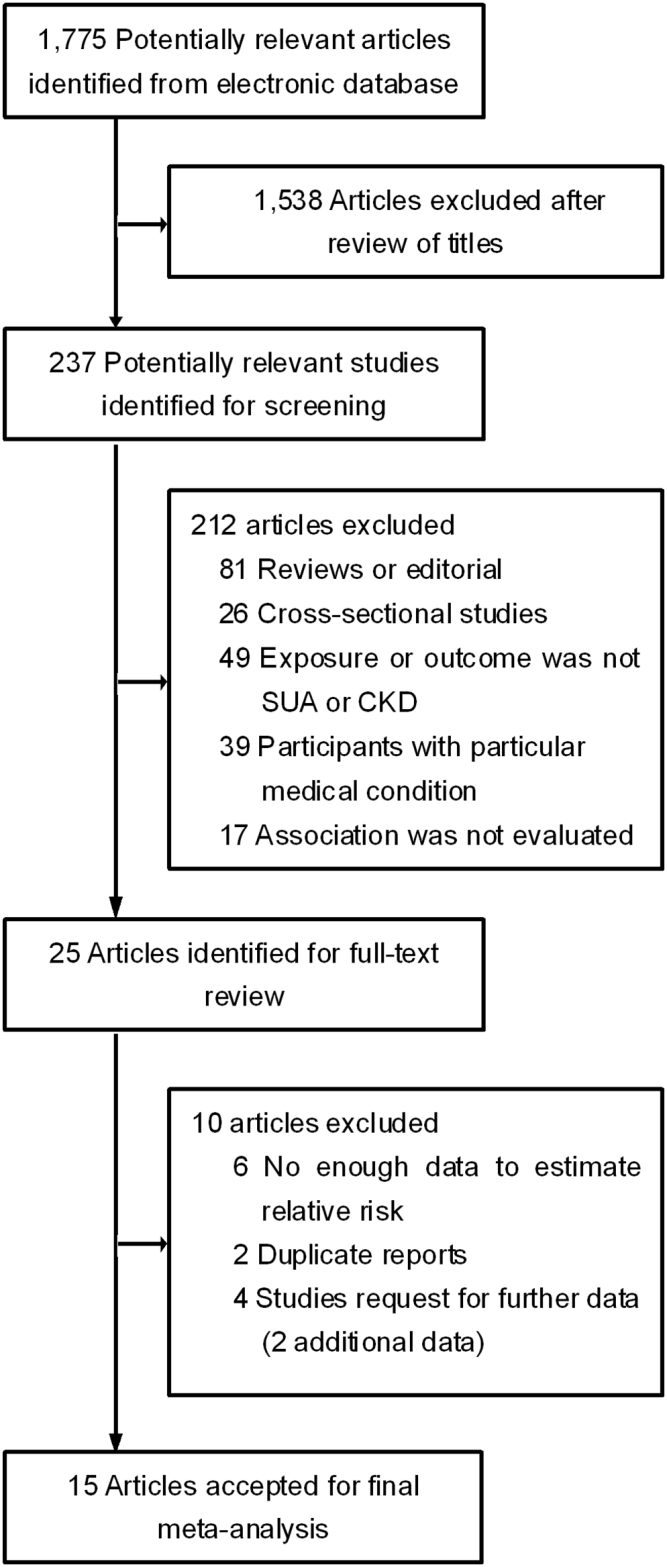
Flow chart of study selection. This flow chart depicts the literature search for longitudinal cohort studies reporting the relationship between the serum uric acid (SUA) level and chronic kidney disease (CKD).

We initially identified 1,775 candidate citations reporting results regarding serum uric acid levels and the risk of CKD. Of these articles, 1,750 were excluded because they were not longitudinal cohort studies or because the exposure or endpoint was not relevant to our analysis after screening the titles and abstracts. After reviewing the full texts of the remaining 25 studies, we identified 15 eligible longitudinal cohort studies [7], [8], [9], [10], [11], [15], [23], [24], [25], [26], [27], [28], [29], [30], [31], including 19 datasets in the analysis; four studies [11], [23], [30], [31] reported the results separately according to gender. One article [10] was identified by checking the reference lists of the retrieved papers. The main reasons for exclusion were the following: duplicate reports using the same population in 2 studies [32], [33], the outcome of interest was not defined as an eGFR <60 ml/min/1.73 m^2^ at the follow-up examinations [34], and absence of risk estimates of the association between CKD and serum uric acid levels in 6 studies. A flow diagram showing the methodology used to select relevant studies is presented in Figure 1.

### Study Characteristics

Fifteen independent cohort studies reporting 99,205 participants and 3,492 incident cases were identified. Four studies were conducted in Western countries, and 11 studies were conducted in Asian countries. The selected studies were published from 2005 through 2013, and the number of subjects per study ranged from 519 to 18,778. Six studies recruited participants from population registries. Seven study populations were from healthy checkup population, and two study population included factory employees. The mean serum uric acid level for the subjects ranged from 4.0 to 6.9 mg/dL, and their mean age ranged from 40.5 to 74.5 years. Two studies exclusively included men, and four studies included women and men. The average follow-up duration ranged from 2.2 to 26.0 years. Most studies adjusted for a wide range of potential confounders for the association between serum uric acid levels and the risk of CKD, including age, sex, eGFR (or SCr), BMI, and other components of metabolic syndrome (plasma glucose, HDL cholesterol, and triglycerols). Nine studies presented results for adjustment to blood pressure, and five presented results for adjustment to hypertension status. Some studies also adjusted for proteinuria and lifestyle (alcohol consumption, smoking, and physical activity). Only two studies considered the effects of diuretic use, and two studies considered the effects of other drugs that influence serum uric acid, such as allopurinol. The characteristics of the selected studies are presented in Table 1.

**Table 1:** Characteristics of the identified cohort studies of serum uric acid levels and the risk of chronic kidney disease.

Author, publicationyear[ref.], country	Study population	Baseline year, follow-up years	Study size, meanage,number ofcases	Baselinehypertension/diabetes(%)	Men(%)	Uricacid(mg/dL)	Cohortdesign	Adjustment forcovariates
Domrongkitc, 2005[15], Thailand	Employees of theElectric GenerationAuthority	1985, 12.0	2967, 42.5 years, 202 cases	8.9/7.4	75.9	5.4	P	Age, sex, BMI, smoking, eGFR, proteinuria, SBP, DBP, diabetes, and total cholesterol
Chonchol, 2007[28], U.S	CardiovascularHealth Study	1989, 6.9	4610, 73.0 years, 240 cases	58.5/16.2	43.0	5.7	P	Age, sex, BMI, medications (allopurinol and diuretics), SCr, SBP, DBP, FPG, HDL cholesterol, triglycerides, ankle-arm index, intima media thickness, hemoglobin, and race
Obermayr, 2008[24], Austria	The Vienna HealthScreening Project	1990, 7.2	17375, 41.9 years, 288 cases	28.2/0.9	53.6	5.2	P	Age, sex, BMI, smoking, regular exercise, eGFR, proteinuria, SBP, DBP, diabetes, FPG, HDL cholesterol, LDL cholesterol, triglycerides, and total cholesterol
Yen, 2009[9], China	TheCommunity-basedcohort	2002, 2.7	519, 74.5 years, no report	46.9/12.8	61.4	M:6.6, W:5.6	P	Age, sex, BMI, smoking, SCr, proteinuria, hypertension, diabetes, total cholesterol, hemoglobin, platelet counts, and albumin
Chien, 2010[27], China	Health-checkpopulation	2003, 2.2	5168, 51.2 years, 190 cases	23.9/12.2	63.3	6.1	P	Age, sex, BMI, history of stroke, proteinuria, DBP, diabetes, and HbA1c
Bellomo, 2010[8], Italy	Healthynormotensiveblood donors	2003, 4.9	824, 43.1 years, 12 cases	0.0/0.0	82.6	4.9	P	Age and eGFR
Sonoda, 2011[26], Japan	Health-checkpopulation	2001, 4.6	7012, 52.8 years, 568 cases	0.0/0.0	64.2	5.3	P	Age, sex, BMI, smoking, eGFR, SBP, FPG, HDL cholesterol, LDL cholesterol, triglycerides, and hemoglobin
Ben-dov, 2011[11], Israel	The Jerusalem LipidResearch Cliniccohort study	1976, 26.0	2449, 48.1 years, 109 cases	18.4/2.0	60.0	M:5.7, W:4.5	P	Age, sex, BMI, smoking, SCr, SBP, fasting plasma glucose, and LDL cholesterol
Wang, 2011[29], China	MJ Longitudinalhealth-checkup-basedPopulationDatabase	1996, 7.0	7488, 40.5 years, no report	16.2/3.4	50.4	M:6.9 W:5.1	R	Age, sex, BMI, alcohol consumption, smoking, medications (allopurinol, antihyperlipidemic), exercise, eGFR, proteinuria, hypertension, diabetes, triglycerides, total cholesterol, LDL cholesterol, HDL cholesterol, and hemoglobin
Kawashima, 2011[31], Japan	Health-check male factory workers	1990, 7.9	1285, 45.7 years, 100 cases	19.8/4.0	100.0	5.8	R	Age, BMI, BP, HDL cholesterol, and FPG
Yamada, 2011[30], Japan	Health-check population	2000, 5.0	12227, 49.1 years, 490 cases	23.0/no report	56.6	M:6.0, W:4.3	R	Age, sex, BMI, alcohol consumption, smoking, eGFR, proteinuria, BP, FPG, and triglycerides
Zhang, 2012[25], China	Population-based	2004, 4.0	1410, 59.1 years, 168 cases	45.1/26.4	48.5	4.9	P	Age, sex, BMI, smoking, eGFR, albuminuria, hypertension, and diabetes
Mok, 2012[23], Korea	The Severance Cohort Study	1994, 10.2	14939, 43.5 years, 766 cases	18.6/4.2	58.1	M:5.8, W:4.0	P	Age, sex, BMI, alcohol consumption, smoking, regular exercise, hypertension, diabetes, and dyslipidemia
Sedaghat, 2013[7], Netherlands	The Rotterdam Study	1990, 6.5	2154, 70.4 years, 249 cases	47.8/11.0	37.7	5.4	P	Age, sex, BMI, alcohol consumption, smoking, medications (diuretics, CCB, and ACEI), eGFR, SBP, diabetes, HDL cholesterol, and total cholesterol
Ryoo, 2013[10], Korea	Employees of Korean companies	2005, 4.0	18778, 41.8 years, 110 cases	15.9/3.1	100.0	6.0	P	Age, BMI, alcohol consumption, smoking, regular exercise, eGFR, hypertension, SBP, diabetes, HOMA-IR, and triglycerides

ACEI, angiotensin-converting enzyme inhibitor; BMI, body mass index; CCB, calcium channel blockers; DBP, diastolic blood pressure; eGFR, estimated glomerular filtration rate; FPG, fasting plasma glucose; HDL, high-density lipoprotein; HOMA-IR, homeostatic model assessment-insulin resistance; LDL, low-density lipoprotein; M, men; P, prospective; R, retrospective; SBP, systolic blood pressure; SCr, serum creatinine; W, women.

### Overall Analyses

**Figure 2:**
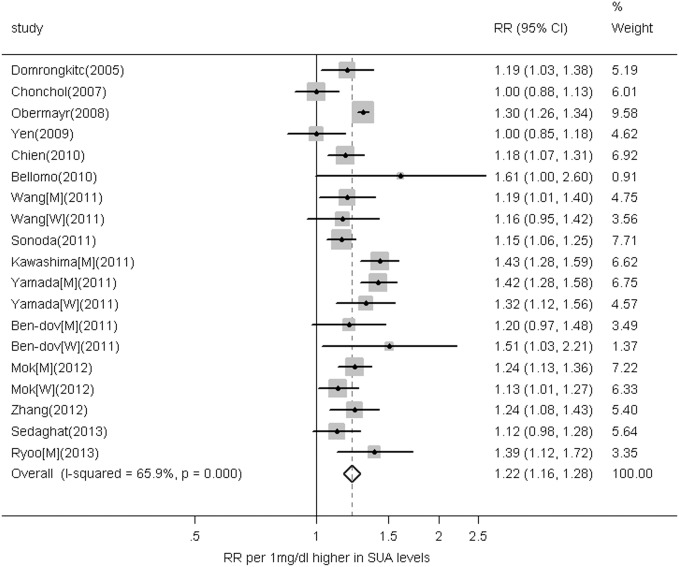
Relative risks for the association between serum uric acid (for a 1 mg/dL increase) and the incidence of CKD. Squares represent study-specific relative risks (the square sizes are proportional to the weight of each study in the overall estimate), horizontal lines represent 95% confidence intervals, and diamonds represent the overall relative risk estimate with its 95% CI.

Of the 15 studies with 19 datasets, 14 datasets had a significant positive relationship between serum uric acid levels and the risk of CKD (Figure 2). Based on a random-effects model meta-analysis, the combined maximally adjusted RR of CKD for a 1 mg/dL higher serum uric acid level was 1.22 (95% CI 1.16–1.28). Significant heterogeneity was observed (I^2^ = 65.9%, P<0.001). No evidence of publication bias was determined with either the Begg’s (P = 0.441) or Egger’s tests (P = 0.222).

### Subgroup and Sensitivity Analyses

**Figure 3:**
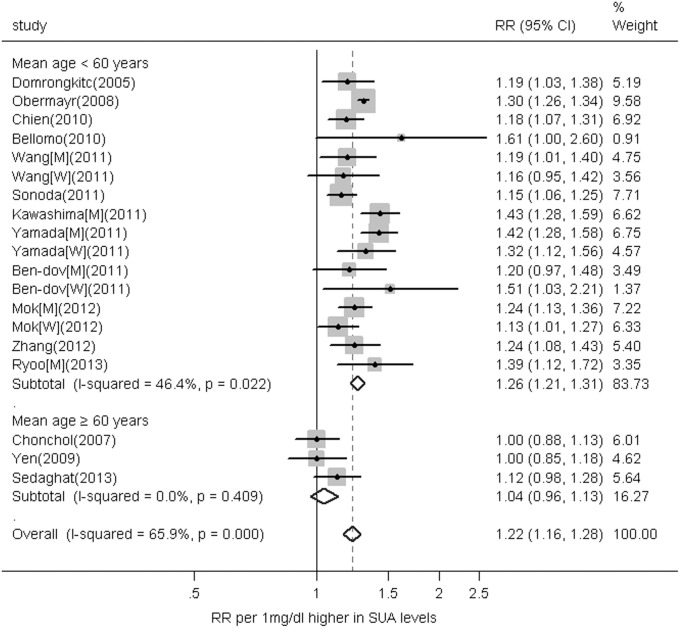
Plot of the RR of CKD associated with a 1 mg/dL increase in the serum uric acid level among individuals according to mean age. The RR was pooled within subgroups with similar mean ages using random-effects model meta-analysis.

We performed stratified analyses to explore the study heterogeneity. The effects of the serum uric acid level on the CKD risk in the subgroup meta-analyses are presented in Table 2. No significant difference in the RRs was observed between subgroups in serum uric acid and CKD analyses stratified according to most of the study-level characteristics, such as gender, study location, follow-up length, measure of association, source of subjects, and mean serum uric acid level. The observed positive association was more pronounced among studies with a mean age <60 years (RR 1.26, 95% CI 1.21–1.31), and low-level heterogeneity was observed (I^2^ = 46.4%, P = 0.022). However, no association was identified among those with a mean age≥60 years (RR 1.04, 95% CI 0.96–1.13), and no heterogeneity among the studies for this age group was observed (I^2^ = 0, P = 0.409, Figure 3). Additionally, the association between serum uric acid levels and CKD significantly differed according to the mean age (P = 0.004 for the interaction).

**Table 2:** Stratified and meta-regression analysis to explore the effects of the study characteristics.

Group	Number of cohorts	Pooled RR (95% CI)^a^	I^2^ (%)	P_h_ b	P_h_ c
Total	15	1.22 (1.16–1.28)	65.9	0.000	
Mean age (years)					
<60	12	1.26 (1.21–1.31)	46.4	0.022	0.004
≥60	3	1.04 (0.96–1.13)	0.0	0.409	
Gender					
Men	6	1.32 (1.23–1.42)	39.6	0.142	0.225
Women	4	1.21 (1.09–1.34)	21.2	0.283	
Mean SUA					
<5.5	10	1.22 (1.15–1.29)	51.3	0.030	0.822
≥5.5	9	1.22 (1.12–1.33)	76.1	0.000	
Study location					
Asian	11	1.23 (1.16–1.30)	61.6	0.004	0.500
Non-Asian	4	1.18 (1.00–1.39)	85.6	0.000	
Duration (years)					
<5	6	1.18 (1.09–1.28)	41.5	0.129	0.845
≥5	9	1.23 (1.15–1.31)	75.2	0.000	
BMI (kg/m^2^)					
<25	11	1.23 (1.16–1.31)	64.7	0.002	0.225
≥25	6	1.17 (1.05–1.31)	50.2	0.074	
Measure of association					
Odds ratio	8	1.20 (1.11–1.30)	80.3	0.000	0.729
Hazard ratio	7	1.23 (1.14–1.32)	49.7	0.063	
Source of subjects					
Health check	10	1.25 (1.16–1.35)	66.8	0.001	0.353
Non-health check	9	1.19 (1.11–1.28)	68.5	0.001	

a The pooled RR of CKD for each 1 mg/dL increase in the SUA within the strata of each study characteristic are indicated.

b P for the heterogeneity within each subgroup.

c P for the heterogeneity between subgroups in the meta-regression analysis. The analysis was based on 15 studies and 19 data points because men and women were included separately for the studies reported by Ben-Dov IZ et al., Mok Y et al., Wang S et al., and Yamada T et al. For the mean age, BMI, mean SUA, and follow-up duration, the P-value was obtained by modeling these variables as continuous variables in the meta-regression analysis.

Furthermore, we conducted sensitivity analyses to evaluate the influence of individual studies on the summary RRs. Compared with the overall analysis, no difference was observed when the analyses were restricted to studies that controlled for metabolic syndrome components (RR 1.21, 95% CI 1.15–1.28) or alcohol consumption (RR 1.24, 95% CI 1.14–1.36). Restricting analysis to studies that controlled for blood pressure or hypertension status yielded a similar RR of 1.23 (1.16–1.32) or 1.18 (1.11–1.26), respectively. The RRs were similar between studies that adjusted for proteinuria and those that did not (1.23, 95% CI 1.15–1.31 vs. 1.21, 95% CI 1.11–1.32). The exclusion of 3 studies [23], [27], [31] that did not adjust for baseline eGFR (or SCr) slightly altered the overall risk estimate (RR 1.21, 95% CI 1.14–1.29), but the heterogeneity remained (I^2^ = 67.1%, P<0.001). Further exclusion of any single study did not materially alter the overall combined results; the RRs for this analysis ranged from 1.21 (1.15–1.27) to 1.24 (1.18–1.29).

## Discussion

In the current meta-analysis of 15 cohort studies, we observed a significant positive association between serum uric acid levels and the incidence of CKD in middle-aged patients. For each 1 mg/dL increment in the serum uric acid level, a 22% increase in the risk of CKD was observed. This finding was consistent and did not differ appreciably according to the study location, follow-up length, mean serum uric acid level, source of subjects, and adjustment for metabolic syndrome components or proteinuria. Notably, the serum uric acid level was independently associated with CKD in middle-aged adults but not in elderly adults.

A recent review by Sedaghat [7] demonstrated a significant association between the serum uric acid level and CKD incidence. Our study has some important strengths compared with that review. We were able to enhance the precision of the risk estimates and perform subgroup and sensitivity analyses to explore the sources of heterogeneity, increasing the clinical relevance of our findings. More importantly, all of the included studies were longitudinal cohort studies, and the subjects with a baseline eGFR <60 mL/min/1.73 m^2^ were excluded. This approach greatly reduces the likelihood of selection bias and reverse causation (possible effects of kidney disease on uric acid). Additionally, the consistency in the positive association between serum uric acid levels and risk of CKD across multiple subgroups in our meta-analysis combined with the lack of publication bias suggest that the association is valid.

Moderate heterogeneity in the RRs was observed across studies, which could be attributed to chance or related to variations in the study population, study methodology, or geographical location. The articles included in the current meta-analyses were generally high quality, pre-specified subgroup analyses, and these factors were not significant sources of heterogeneity. Conversely, a more pronounced association was observed in the subgroup with a mean age <60 years compared with the subgroup with a mean age ≥60 years, in which the association between serum uric acid level and CKD disappeared. Meanwhile, the mean age affected the association between serum uric acid levels and CKD, and the test for interaction was significant. The significant decrease in the heterogeneity in the subgroup analyses according to the mean age suggests that age might, at least partially, contribute to the observed increased risk of CKD in middle-aged patients compared with elderly individuals. Age may be an effect modifier of the serum uric acid level and CKD association. Most of the included studies adjusted for age, but none of the studies explored the influence of age on the association between serum uric acid level and CKD risk. Several hypotheses might explain this finding in the elderly population. First, the baseline demographic and clinical characteristics of the participants in three studies [7], [9], [28] with patients ≥60 years of age indicated the presence of significantly higher proportions of chronic comorbidities, such as hypertension and diabetes, compared with other studies. Hypertension and diabetes are the most common causes of CKD [35]. These factors may attenuate the strength of the relationship between serum uric acid levels and CKD. Second, it is difficult to evaluate the SCr levels of elderly people because their muscle mass is unpredictable at any given level of SCr [36]. Potential heterogeneity in renal function exists at relatively normal-appearing SCr and eGFR levels, and a definition of incident CKD that depends on a final eGFR of <60 mL/min/1.73 m^2^ may bias the results toward a null finding [34]. Finally, the length of the follow-up period and the sample size may mask the effect of uric acid on incident CKD for relatively short observation periods or small sample sizes.

Elevated serum uric acid concentrations are correlated with various metabolic profiles [6] and healthy lifestyles [37], such as moderate-to-heavy alcohol consumption and metabolic syndrome components. Consequently, the potential influences of these factors should be considered when interpreting the results. Our sensitivity analysis indicated that a positive association persisted and remained significant even when analyses were limited to studies that adjusted for metabolic syndrome or alcohol intake. The existing evidence from cohort studies supports an independent, exacerbating effect of serum uric acid on CKD.

Recent experimental metabolic studies consistently indicate that uric acid may have a causative role in the development of CKD. Although serum uric acid is believed to possess antioxidant activity in the extracellular environment, uric acid appears to exert various deleterious effects once it enters cells [38]. Uric acid enters vascular smooth muscle cells and activates intracellular protein kinases and nuclear transcription factors, resulting in a proliferative phenotype with the production of cyclooxygenase-2 and monocyte chemoattractant protein-1 and the activation of the rennin-angiotensin system [39]. Increased uric acid inhibits the proliferation and migration of endothelial cells and the secretion of nitric oxide [40], contributing to endothelial dysfunction. Additionally, uric acid can promote proinflammatory mechanisms and induce NADPH oxidase stimulation, thereby promoting mitochondrial dysfunction. In animal studies [41], hyperuricemia results in the development of glomerular hypertension in association with elevated renal vascular resistance and impaired peritubular circulation. Uric acid can also induce an epithelial-to-mesenchymal transition in renal tubular cells [42], which might serve as one of the earliest components of renal fibrosis. In addition to the direct role of uric acid in CKD,it is not implausible that uric acid plays a detrimental role through established risk factors for CKD, such as blood pressure [38]. Although the blood pressure or hypertension status was adjusted in the analyses of the association between uric acid and CKD in most studies, only one study [7] evaluated the impacts of blood pressure on this association by dividing participants into two groups according to complicated with hypertension or not. This study identified more pronounced associations in hypertensive individuals than normotensive individuals. These data indicated that the effect of blood pressure on the relationship between uric acid and CKD required further exploration. Furthermore, evidence from randomized controlled studies suggests that uric acid-lowering therapy with allopurinol may retard the progression of CKD [12], although the available evidence is limited to a small number of single-center studies with suboptimal methodology.

The limitations of this meta-analysis must be considered. First, the observational nature of the cohort studies included in our analysis means that there could be residual factors, although differences in the mean age appear, at least in part, to explain this finding. Few studies considered significant confounders that influence serum uric acid, such as dietary factors [43] or drug history (e.g., diuretic agents and allopurinol). These confounders could modify the association between the serum uric acid levels and risk of CKD. In the case of allopurinol, if uric acid were directly toxic to the kidney or a marker of kidney risk, the lack of allopurinol data would likely have biased the results. High consumption of purine-rich food has been associated with the development of hyperuricemia [44]. Moreover, diet can contribute to the risk of developing CKD [45]. These factors may confound the association between uric acid levels and CKD. Second, misclassification of CKD in the original studies may have affected the results. Some studies used a single baseline uric acid measurement to predict the patient outcome, and the eGFR was not re-evaluated when CKD was diagnosed. This type of misclassification would bias the studies toward a lack of an association. Third, the background population is not representative of the community because the subjects in some studies were recruited from individuals who participated in a preventive medical examination center evaluation of their health. However, the consistency of the finding of an increased risk of CKD in individuals with higher serum uric acid levels in both health-check and community-based populations suggests that the association is valid. Fourth, although the term “incident CKD”, which was artificially defined as eGFR <60 ml/min/1.73 m^2^ (eGFR categories G3a-G5) and did not include albuminuria, was used in most of the studies included in our meta-analyses, the studies are actually predicting “incident eGFR <60 ml/min/1.73 m^2^ CKD” according to the latest guideline for CKD definition and classification [46], [47]. Finally, because our meta-analysis was based on published studies, publication bias could be of concern. Nevertheless, we found no evidence of such bias in this meta-analysis.

In conclusion, our meta-analysis of cohort studies provides strong evidence that high serum uric acid levels increase the risk of CKD independent of conventional metabolic risk factors. Age may be a potential confounding factor in the risk estimates. Direct evidence from future randomized, high-quality trials is warranted to definitively clarify the relationship between serum uric acid levels and CKD.

## Supporting Information

Checklist S1 **PRISMA 2009 checklist.**
(DOC)
